# FNR Regulates the Expression of Important Virulence Factors Contributing to the Pathogenicity of Avian Pathogenic *Escherichia coli*

**DOI:** 10.3389/fcimb.2017.00265

**Published:** 2017-06-23

**Authors:** Nicolle L. Barbieri, Jessica A. Vande Vorde, Alison R. Baker, Fabiana Horn, Ganwu Li, Catherine M. Logue, Lisa K. Nolan

**Affiliations:** ^1^Department of Veterinary Microbiology and Preventive Medicine, College of Veterinary Medicine, Iowa State UniversityAmes, IA, United States; ^2^Departamento de Biofísica, Universidade Federal do Rio Grande do SulPorto Alegre, Brazil; ^3^Department of Infectious Disease, College of Veterinary Medicine, University of GeorgiaAthens, Georgia

**Keywords:** APEC, FNR, virulence regulation, plasmid-linked virulence genes, outer membrane protein

## Abstract

Avian pathogenic *Escherichia coli* (APEC) is the etiologic agent of colibacillosis, an important cause of morbidity and mortality in poultry. Though, many virulence factors associated with APEC pathogenicity are known, their regulation remains unclear. FNR (fumarate and nitrate reduction) is a well-known global regulator that works as an oxygen sensor and has previously been described as a virulence regulator in bacterial pathogens. The goal of this study was to examine the role of FNR in the regulation of APEC virulence factors, such as Type I fimbriae, and processes such as adherence and invasion, type VI secretion, survival during oxidative stress, and growth in iron-restricted environments. To accomplish this goal, APEC O1, a well-characterized, highly virulent, and fully sequenced strain of APEC harboring multiple virulence mechanisms, some of which are plasmid-linked, was compared to its FNR mutant for expression of various virulence traits. Deletion of FNR was found to affect APEC O1's adherence, invasion and expression of *ompT*, a plasmid-encoded outer membrane protein, type I fimbriae, and *aatA*, encoding an autotransporter. Indeed, the *fnr*^−^ mutant showed an 8-fold reduction in expression of type I fimbriae and a highly significant (*P* < 0.0001) reduction in expression of *fimA, ompT* (plasmid-borne), and *aatA*. FNR was also found to regulate expression of the type VI secretion system, affecting the expression of *vgrG*. Further, FNR was found to be important to APEC O1's growth in iron-deficient media and survival during oxidative stress with the mutant showing a 4-fold decrease in tolerance to oxidative stress, as compared to the wild type. Thus, our results suggest that FNR functions as an important regulator of APEC virulence.

## Introduction

*Escherichia coli* is an important gastrointestinal inhabitant. It is present in the microbiota of humans and other warm blooded animals (Yan and Polk, 2004). *E. coli* is usually a non-pathogenic bacterium; however, some strains of *E. coli* have acquired virulence factors via horizontal genetic transfer to become pathogenic. Virulence factors confer on bacteria the ability to adapt to new niches, exploit resources available there and cause disease. Though Extraintestinal Pathogenic *E. coli* (ExPEC) strains may exist in the gut without causing disease, they have the capacity to disseminate and colonize host niches beyond the gut such as the urinary tract, bloodstream, and central nervous system, resulting in localized or systemic infection and disease, depending on the panoply of virulence mechanisms they possess and express during infection (Wiles et al., 2008).

The ExPEC of poultry, Avian Pathogenic *Escherichia coli* (APEC), causes colibacillosis, a disease which is a significant economic burden on all facets of the poultry industry (Nolan et al., 2013). APEC infection is manifested as a variety of conditions in birds including colisepticemia, airsacculitis, swollen head syndrome, and cellulitis, which may result in poor egg quality, decreased production, condemnations, and increased morbidity and mortality (Schouler et al., 2012; Nolan et al., 2013). Though much progress has been made in understanding the virulence factors contributing to the pathogenesis of these diseases, little is known about how APEC virulence is regulated during infection. Perhaps, the most common virulence traits among APEC include the ability to adhere to host tissues, survive within host fluids, and resist the host immune defenses (Ewers et al., 2007; Johnson and Nolan, 2009).

More than 250 transcription factors are known to regulate gene expression in *E. coli*. Some of these factors are operon-specific while others, known as global regulators, coordinate the expression of numerous promoters in response to specific environmental cues (Martinez-Antonio and Collado-Vides, 2003). Although, many virulence factors are known to be associated with APEC pathogenicity, the regulation of their expression is still not fully understood.

One candidate regulator that might be important in the regulation of APEC virulence is FNR (fumarate and nitrate reduction), a well-known global regulator of oxygen utilization in non-pathogenic *E. coli*. It is activated under anaerobic conditions and is important in controlling up to 125 genes in non-pathogenic *E. coli* (Kiley and Beinert, 1999) and over 300 genes in Uropathogenic *E. coli* (UPEC, a type of ExPEC), *Shigella flexneri* and *Salmonella* (Fink et al., 2007; Marteyn et al., 2010; Barbieri et al., 2014). Previous studies from our lab investigated FNR associated regulation in UPEC and demonstrated regulation of a number of virulence genes (Barbieri et al., 2014) and because of our previous observations we wanted to assess the potential role of FNR in another ExPEC—avian pathogenic *E. coli*. However, to our current knowledge there are no other studies of FNR regulating APEC genes. Therfore, we hypothesize that FNR may regulate a number of genes or mechanisms that contribute to APEC's ability to survive in its host and cause disease. Using bioinformatic analysis, potential targets of FNR regulation were examined and a series of genes selected for this study including *ChuA, OmpT, mig-14, EstA, Vrg, aatA*, and *fim*.

ChuA is an outer membrane protein responsible for heme uptake in *E. coli* (Nagy et al., 2001) and one of many mechanisms by which *E. coli* acquires iron for electron transport processes (Payne, 1993). Outer Membrane Protein T (OmpT), is encoded by *ompT* associated with adherence and antimicrobial resistance (Thomassin et al., 2012). APEC O1 contains two copies of this gene, one being chromosomally located, while the second is borne on the pAPEC-O1-ColBM plasmid (Johnson et al., 2007). Another plasmid-linked virulence factor is *aatA*, which encodes a novel autotransporter that mediates adherence to chicken fibroblasts and contributes to virulence (Li et al., 2009).

Pathogenic bacteria like APEC must cope with oxidative stress imposed by the innate immune system via macrophages during infection (Schlosser-Silverman et al., 2000; Missall et al., 2004). *E. coli* are aided in their resistance to macrophage-induced destruction by the “macrophage induced gene” or *mig*, which helps to protect *E. coli* from DNA damage (Schlosser-Silverman et al., 2000).

During infection, APEC may also encounter antimicrobial agents, such as the macrolide erythromycin (Nolan et al., 2013). EtsA is a plasmid-linked putative macrolide efflux pump that was first described in APEC O1 by Johnson et al. (2006). The VgrG subunit contributes to the formation of the type VI secretion system (T6SS) needle and is likely released from the system once the target cell is punctured, FNR is involved in its regulation (Silverman et al., 2012).

The purpose of this study was to determine if these identified genes linked to APEC or *E. coli* virulence or resistance are regulated by the global regulator FNR under aerobic conditions.

## Materials and methods

### Bacterial strains and culture conditions

The wild-type bacterial strain used in these studies was APEC O1 (Johnson et al., 2007). APEC O1 is an O1:K1:H7 strain that was originally isolated from the lung of a chicken clinically diagnosed with colisepticemia. The O1 serogroup is one of the more commonly occurring serogroups among APEC and human uropathogenic *E. coli*. It is considered to be highly virulent for chickens.

Strains and plasmids used in this study are listed in Table S1 in the Supplementary Material. Aerobic growth was achieved by orbital shaking at 180 rpm in Pyrex glass bottles with 1/3 of volume used. For genetic manipulations, all *E. coli* strains were grown routinely in Luria Bertani (LB) broth. Selective antibiotics and IPTG were added when necessary at the following concentrations: ampicillin (Amp), 100 μg ml^−1^; kanamycin (Kan), 50 μg ml^−1^; chloramphenicol (Chl), 25 μg ml^−1^; IPTG, 0.1 mM (Cai et al., 2013; Barbieri et al., 2014).

### Recombinant DNA techniques

PCR, DNA ligation, electroporation, and DNA gel electrophoresis were performed as described by Sambrook and Rusell (2001) and Barbieri et al. (2014) unless otherwise indicated. All oligonucleotide primers were purchased from Integrated DNA Technologies (IDT; Coralville, IA) and are listed in Table S2 in the Supplementary Material. All restriction and DNA-modifying enzymes were purchased from New England BioLabs (NEB; Ipswich, MA) and used according to the supplier's recommendations. Recombinant plasmids, PCR products, and restriction fragments were purified using QIAquick PCR purification kits or MinElute gel extraction kits (Qiagen, Valencia, CA) as recommended by the supplier. DNA sequencing was performed at Iowa State University's DNA facility. Deletion mutants were constructed using the bacteriophage lambda red recombinase system described by Datsenko and Wanner (Datsenko and Wanner, 2000; Barbieri et al., 2014).

Chromosomal transcriptional *lacZ* fusions were introduced into an APEC O1 strain in which the original *lacZYA* genes were deleted. The homologous recombination constructs used the suicide plasmid pVIK112 carrying a fragment of the complete 5′ region or 3′ region of the target gene, leaving the target functional (Kalogeraki and Winans, 1997; Cai et al., 2013). For complementation, the coding sequences of the genes plus their putative promoter regions were amplified from APEC O1 and independently cloned into pGEN-MCS (Lane et al., 2007) using *EcoRI* and *SalI* restriction sites.

### Impact of FNR on APEC's adherence and invasion

Cell adherence and invasion assays were performed as previously described (Vigil et al., 2012). HeLa (epithelial cells; de Pace et al., 2010; Stacy et al., 2014; Matter et al., 2015; Verma et al., 2015) and J774 (murine macrophages; Bastiani et al., 2005) cells, rather than avian origin cells, were used in these studies because they are well-known for their utility in adherence and invasion assays in APEC studies (Vigil et al., 2012). The cell lines were cultured in DMEM Medium (GIBCO) containing 10% fetal bovine serum (FBS; GIBCO), at 37°C and 5% CO_2_. Cells were plated in sterile 24-well-plates with 1 × 10^5^ cells/well 48 h prior to each experiment. Wild-type APEC O1, mutant and complemented strains were cultured statically in LB medium for 16 h. Before infection, two wells of cultured cells were trypsinized, and the cells counted to estimate the cell number per well. Cells were washed once with 1 ml PBS and then were exposed to bacteria, washed once with PBS, at a multiplicity of infection (MOI) of 10 in a 1 ml volume of media. The 24-well-plates were centrifuged (500 g for 5 min) and incubated for 1 h (adherence/internalization), 4 h (invasion/survival) or 24 h (persistence).

#### For the adherence/internalization assays

Bacterial-exposed cells were washed four times with 1 ml of sterile PBS and then lysed with 1 ml 0.1% Triton-X 100 for 10 min at room temperature. Serial dilutions of cell suspensions were spread onto MacConkey agar plates, and CFU counts were obtained following overnight growth at 37°C.

#### For the invasion/survival and persistence assays

After 1 h of incubation, infected cells were washed four times with 1 ml of sterile PBS and then re-incubated with fresh DMEM Medium containing 100 μg/ml gentamicin for 3 or 23 h of incubation. At 4 and 24 h post-infection, cells were washed four times with 1 ml of sterile PBS and then lysed with 1 ml of 0.1% Triton-X 100 for 10 min at room temperature. Serial dilutions of cell suspensions were spread onto MacConkey agar plates, and CFU counts were obtained after overnight growth at 37°C. The input dilution of bacteria was also plated to determine the CFU count for each inoculum.

#### To visualize the association between bacteria and cell lines

Cells were plated on glass coverslips in 24-well-plates (1 × 10^5^ cells per well) and infected with bacteria at an MOI of 10 CFU per cell as described above. After 1, 4, or 24 h post-infection, cells were fixed *in situ* with 3.7% formaldehyde in PBS for 10 min at room temperature and stained with Giemsa [12 drops of Giemsa's solution (Merck, Germany) in 10 ml distilled water] for 20 min at room temperature. Samples were then washed with water and observed under a light microscope at x1,000.

### Bioinformatics analysis

**Figure 1:**
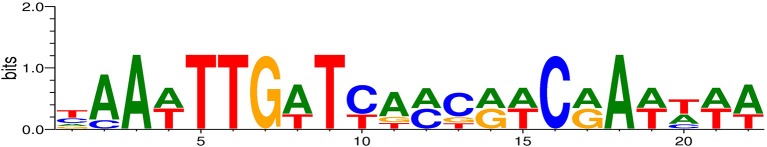
Logograph of the information matrix obtained from the consensus alignment of the FNR motif sequences for APEC O1. The total height of each column of characters represents the amount of information for that specific position, and the width of each character represents the frequency of each nucleotide. We predicted the binding sites on the APEC O1 chromosome (NC_008563.1), on the plasmid p-APEC-O1-ColBM (NC_009837.1), and on the plasmid p-APEC-O1-R (NC_009838.1).

The information matrix for the generation of the FNR logograph was produced by using the alignment of the *E. coli* FNR binding sequences as described previously by Fink et al. (2007). To account for differences in nucleotide usage or slight variations in consensus sequences, a second alignment was built for *E. coli* APEC O1 using the regions of the homologous genes originally used to build the *E. coli* information matrix. The alignment was used to prepare a new information matrix using the Patser software (version 3d). A graphical representation (Figure 1) of the matrices through a logograph was obtained with Weblogo software (version 2.8.1).

To further confirm FNR's contributions to the regulation of APEC O1's virulence, we searched APEC O1's genomic sequence for putative FNR binding sites using an the *E. coli* APEC O1 specific logograph (Figure 1). Five hundred and sixteen binding sites were predicted in the APEC O1 chromosome (NC_008563.1), and 19 binding sites were predicted in APEC O1's virulence plasmid, pAPEC O1-ColBM (NC_009837.1). Predicted binding sites were found upstream of most known virulence genes and operons, including such plasmid genes as those encoding AatA, a putative macrolide efflux pump, resistance to oxidative stress and OmpT, and encoded in the chromosome Type I Fimbriae, ChuA (heme receptor), and T6SS (Table 1). These seven genes were chosen for further study as they have been previously associated with a gain in virulence of APEC or other pathogenic *E. coli* under experimental conditions (Schlosser-Silverman et al., 2000; Johnson et al., 2006; Sabri et al., 2006; Johnson and Nolan, 2009; Li et al., 2009; de Pace et al., 2010; Gao et al., 2012; Thomassin et al., 2012) and the effects of a global regulator on their expression could be measured under laboratory conditions. These genes and their relationship to FNR regulation were subjected to further analysis as described below.

**Table 1:** FNR binding predicted *in silico*.

**Gene name**	**ORF ID**	**Gene description**	**Start**	**End**	**Strand**	**PWM score**	**SEP score**	**Sequence**	**ATG-distance**
*mig-14*	APECO1_O1CoBM188	Macrophage induced gene	162,820	162,833	+	7.11	−5.91	CTGATTATGATAAA	−47
*aatA*	APECO1_O1CoBM96	Autotransporter adhesin	81,838	81,851	−	7.21	−6.26	ATGTTTTTTCTCAA	−79
*ompT*	APECO1_O1CoBM192	Outer membrane protein plasmid	165,359	165,372	+	7.77	−5.94	TTGTTTTTGATAAA	−64
*etsA*	APECO1_O1CoBM197	Putative macrolide efflux pump	169,537	169,550	−	7.01	−6	TTGGAATTAATCAT	−84
*chuA*	APECO1_2948	Outer membrane iron receptor	3,948,831	3,948,818	−	7.09	−5.95	TTGTAATGTTTCAA	−66
*fimI*	APECO1_2117	Type 1 fimbriae	4,922,969	4,922,982	−	6.79	−6.45	TTGAAACTACTCAC	−40
*vgrG*	APECO1_1758	Type VI secretion protein	263,090	263,103	+	6.72	−6.77	ATGGTTTTCCGCAA	−78

### β-galactosidase assays for expression analysis

Overnight LB cultures of *E. coli*, containing the gene of interest fused to lacZ, were washed with PBS once and then diluted 1:100 in LB or indicated media and grown at 37°C. For analysis of *fim*, a single colony was picked from an LB agar plate and inoculated into 5 ml of LB and allowed to grow overnight (pre-inoculum); then 200 μl were inoculated into a new bottle of 5 ml (glass Pyrex bottle 15 ml) of LB and incubated aerobically for 48 h at 37°C without shaking (Barbieri et al., 2014). For analysis of *chuA*, a single colony was picked from an LB agar plate, inoculated into 5 ml of LB medium with 2,2′-dipyridyl, an iron chelator, and allowed to grow for 2 h.

For analysis of *ompT* and *aatA*, cultures were grown in LB with shaking (150 rpm) until an optical density (OD) at 600 nm (OD_600_) 0.5 was reached. For analysis of *mig*, and *ets*, a single colony was taken from a LB agar plate, inoculated into 5 ml of LB medium at 37°C and grown overnight aerobically (Müller et al., 2009). These cultures were collected when the OD_600_ reached 0.4, diluted 1:10 in Z Buffer (Na_2_HPO_4_ 0.06M, NaH_2_PO_4_ 0.04M, KCl 0.01M, MgSO_4_.001M, β-mercaptoethanol 0.05M, H_2_O, 7.0 pH), and assayed for β-galactosidase activity using *ortho*-nitrophenyl-β-galactoside (ONPG) as the substrate, as described previously (Miller, 1972). β-galactosidase activity was reported as the mean of three biological replicates and four technical replicates.

### Electrophoretic mobility shift assays (EMSAs)

We used the (FNRD154A)_2_ protein variant for the EMSAs assays because it displayed DNA-binding affinities and transcriptional regulatory activities with various FNR-dependent promoters a gift from Dr. Aixin Yan (Shan et al., 2012). Protein expression was performed as described previously (Lazazzera et al., 1993). Briefly, *E. coli* BL21 with pET28a-(FNRD154A)_2_ was grown in 200 ml of LB medium for 16 h at 25°C with 0.1 mM IPTG. To study the binding of FNR to the DNA probe, EMSAs were performed as described elsewhere (Shen et al., 2011; Shan et al., 2012). Briefly, the FNR-His6 fusion protein was purified to homogeneity using Ni-nitrilotriacetic acid spin columns (Lazazzera et al., 1993) and dialyzed with binding buffer. DNA probes were amplified using specific primers and purified using a Qiagen MinElute gel extraction kit. EMSAs were performed by adding increasing amounts of purified (FNRD154A)_2_-His6 fusion protein (0–15 ng) to the DNA probe (300 ng) in binding buffer (20 mM Tris, 50 mM NaCl, 40 mM EDTA, 4 mM DTT, 10% glycerol, pH 6.8) for 30 min at 37°C. The reaction mixtures were then subjected to electrophoresis in a 6% polyacrylamide gel in 0.5 TBE buffer (44.5 mM Tris, 44.5 mM boric acid, 1 mM EDTA, pH 8.0) at 200 V for 45 min. The gel was stained in ethidium bromide solution for 20 min., and the image recorded.

### Phenotypic analyses

#### Impact of FNR on APEC O1's type 1 fimbrial activity

To assess FNR's regulation of Type 1 fimbriae, an agglutination assay was used. Tests for mannose-sensitive agglutination were performed as described by Hagberg et al. (1981). Bacterial strains were grown at 37°C in LB broth without shaking for 48 h, then the culture was pelleted by centrifugation and the pellet diluted with PBS to an OD_600_ of 5.0. Then, overnight grown cultures of *Saccharomyces cerevisiae* cells were washed three times in PBS and re-suspended in PBS (10% w/v) prior to use. The yeast cell suspension was mixed on a glass slide with PBS or the bacterial suspension of interest, in the presence and absence of D-mannose (1%). Agglutination was read after 10 min at room temperature. The strength of the agglutination was determined by titering of serial 2-fold dilutions of the bacterial suspensions in PBS (Hagberg et al., 1981).

#### Impact of FNR on APEC O1's growth in iron-deficient medium

To assess FNR's impact on *chuA*'s contribution to APEC O1's growth in iron-deficit environments, APEC O1 and its mutants were grown in LB broth statically overnight. Then, 1 ml of this culture was added to 100 ml of LB media, with or without the iron chelator, 12.5 μM 2,2′-dipyridyl added. The OD_600_ of the culture was measured every 30 min for at least 330 min and recorded (Gao et al., 2012). The assay was repeated three times.

#### Impact of FNR on APEC O1's T6SS activity

To determine if FNR contributes to the regulation of T6SS expression, a *Dictyostelium* plaque assay was performed according to the method of Pukatzki et al. (2006) with slight modifications. Bacterial cells were grown to an optical density of 0.5 at OD_600_ in LB medium, diluted to 1 × 10^8^ CFU/ml in PBS, and plated onto SM5 agar plates (glucose 2 g/l; bacto peptone 2 g/l; yeast extract 0.2 g/l; magnesium sulfate heptahydrate 0.1 g/l; monobasic potassium phosphate 1.9 g/l; bibasic potassium phosphate 1.0 g/l; and agar 15 g/l; pH 6.5). Five microliters of 2-fold serial dilutions of the amoeba were spotted onto the plate and allowed to dry. The plates were incubated in the dark at 22°C for 3–5 days. The lowest concentration of amoeba that permitted bacterial survival was determined to be the minimal inhibitory concentration (MIC). Amoeba concentrations varying from 1 × 10^1^ to 1 × 10^5^ cells/spot were used in the assay.

#### Impact of FNR on APEC O1's antimicrobial resistance

Using an LB agar plate, wild type (APEC O1), ΔFNR, and APEC O1 ΔFNR +pGEM-FNR strains were streaked for isolation and incubated at 37°C for 18 h. For analysis of the resistance to antimicrobial conferred by *ets, mig*, and *ompTs*, minimum inhibitory concentration (MIC) in the presence of various agents were determined using broth microdilution (Wiegand et al., 2008). Fifty microliters of LB broth was added to wells 1–11 of a 12 column 96-well-plate (Falcon 351177 microtest U-bottom polystyrene with low evaporation lids). For *ets* analysis, a 2-fold serial dilution of erythromycin was added to each row of wells. For *mig* analysis, a 2-fold serial dilution of H_2_O_2_ was added to each row of wells. For *ompT* analysis, a 2-fold serial dilution of LL-37, Polymyxin B and Lysozyme were added to each row of wells. Fifty microliters of bacteria of an optical density of 0.5 at 600 nm was diluted to 5 × 10^5^ CFU/ml in LB medium and portioned into rows of wells. A sterile cover was placed over the completed 96-well-plate and incubated at 37°C for 18 h without shaking. The lowest concentration of erythromycin, LL-37, Polymyxin B, Lysozyme or H_2_O_2_ containing no visible growth was identified as the MIC. The antimicrobials used were: LL-37 (512 μg/ml in water; Anaspec inc.), Polymyxin B (512 μg/ml in water; Sigma), Lysozyme (20 mg/ml in water; Sigma), Erythromycin (256 μg/ml in water; Acros Organic), and Hydrogen Peroxide (30% in water; Fisher BioReagents) The antimicrobial concentration test ranges were: LL-37 (128 to 0.002 μg/ml), Polymyxin B (128 to 0.002 μg/ml), Lysozyme (5,000 to 2.4 μg/ml), Erythromycin (64 to 0.062 μg/ml), and Hydrogen Peroxide (15 to 0.013%; diluted from the original new closed bottle).

## Results

### Deletion of *fnr* reduced APEC O1's adherence and invasion

**Figure 2:**
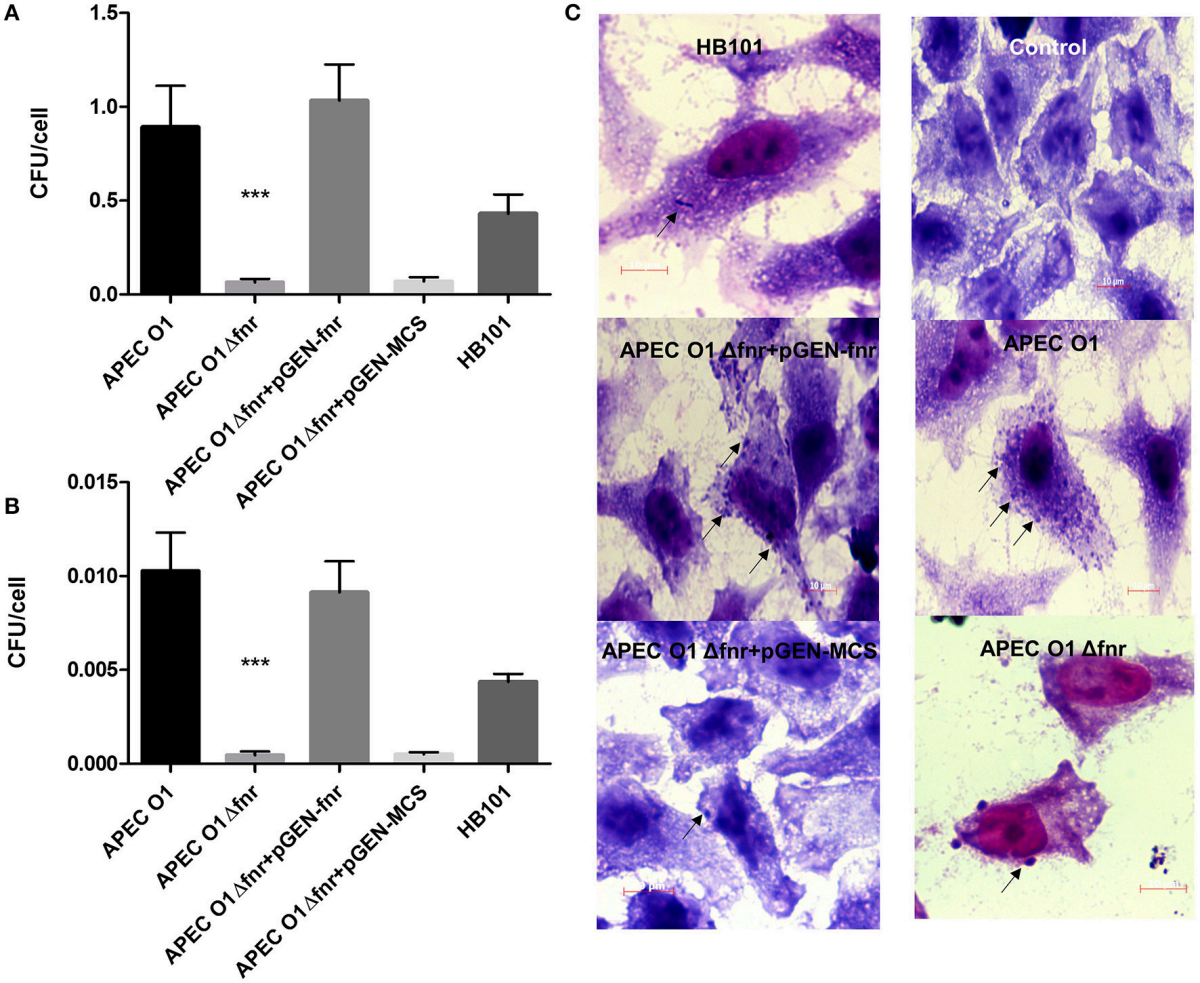
Adherence and invasion of HeLA cells. HeLa cells were infected at MOI of 10 CFU/cell. For adherence assays **(A)**, cells were lysed at 1 h post-infection, and the extracts plated. For the invasion assays **(B)**, at 1 h post-infection, cells were washed and incubated for further 3 h in the presence of gentamicin. Cells were then lysed, and extracts plated. *E. coli* HB101 was used as a negative control. The values shown are means plus standard deviations. Significant differences are indicated by asterisks (^***^P < 0.0001). At 4 h post-infection, the association of bacteria with HeLa cells was visualized by Giemsa staining **(C)**. Arrows indicate bacteria associated with the HeLa cells. Magnification is 1000×.

Successful colonization of the respiratory tract relies on APEC's ability to adhere to and invade host cells and tissues. To determine the effect of *fnr* mutation on APEC O1's adherence and invasion of epithelial cells *in vitro*, cultured HeLa cells were infected with the WT, the *fnr* mutant, the complemented (pGEN-FNR) and *fnr* mutant containing the empty vector (pGEN-MCS) strains. Results showed that deletion of *fnr* significantly decreased APEC's adherence to (*P* < 0.01) and invasion of (*P* < 0.01) HeLa cells (Figures 2A–C). The WT strain displayed an association of 0.893 bacteria per cell; whereas, the *fnr* mutant displayed an association of 0.063 bacteria per cell, a 14-fold reduction in host cell adherence. Further, the WT strain showed an invasion of 0.01 bacteria per cell; whereas, the *fnr* mutant showed an invasion of 0.0004 bacteria per cell. When expressed as percent invasion (calculated by dividing the number of intracellular bacteria at 4 hpi. by the number of the corresponding adherent bacteria at 1 hpi. and taking the amount of associated bacteria as 100%), we found that 1.15% of the adherent WT cells actually invaded cells, but only 0.71% of the adherent *fnr* mutant strains were able to invade these cells. Thus, the mutant strain showed a 0.38-fold reduction in invasion of epithelial cells as compared to the WT. Complementation of the mutant by reintroduction of *fnr* restored adherence and invasion to WT levels (Figures 2A–C). These results suggest that mutation of *fnr* reduces adherence and invasion of APEC to HeLa cells.

### Deletion of *fnr* reduced APEC O1's persistence in cultured J774 cells

**Figure 3:**
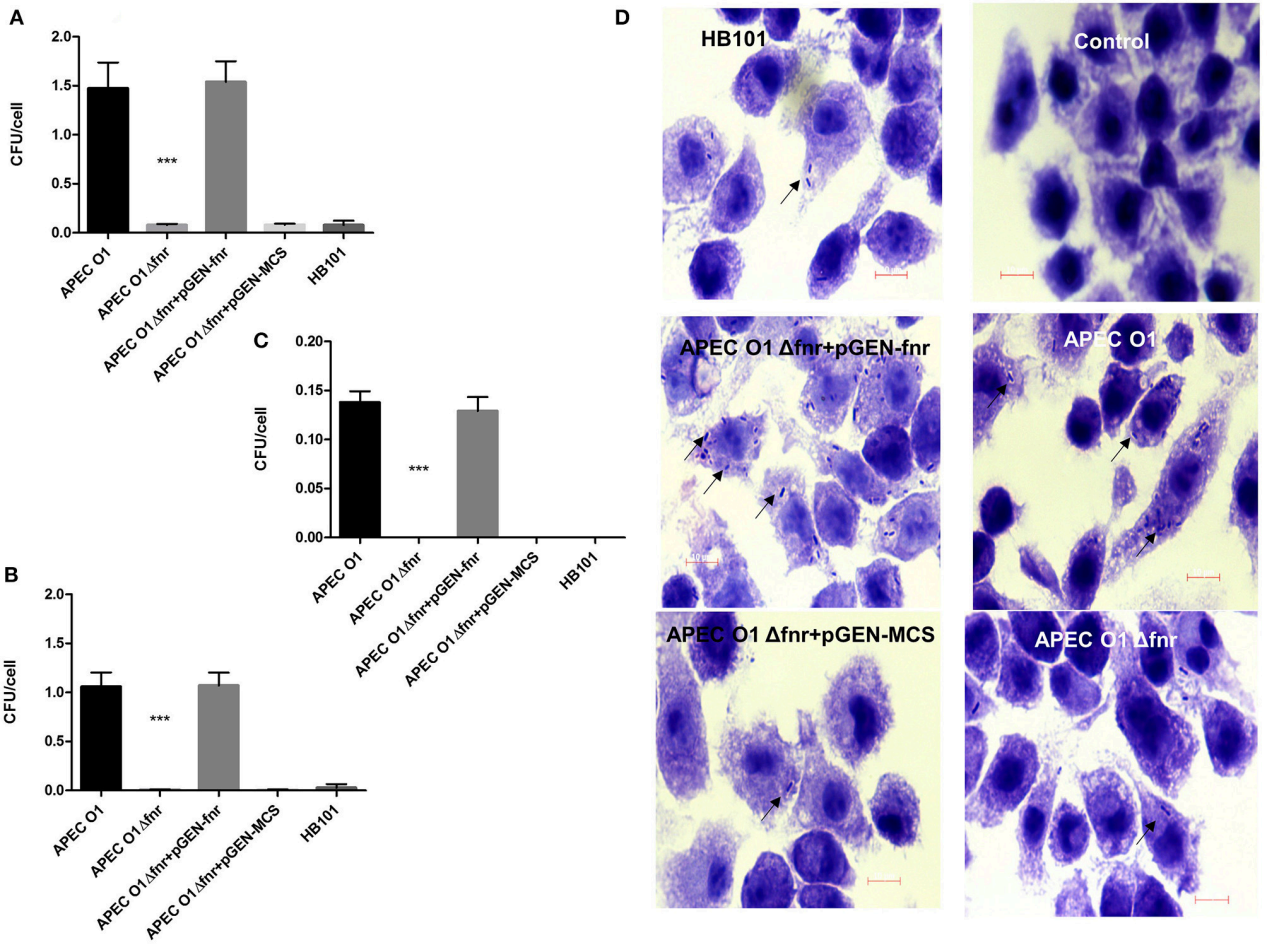
Survival and persistence in macrophages. J774 cells were infected at MOI of 10 CFU/cell. At 1 h post-infection **(A)**, cells were washed with PBS and incubated for more 3 h with gentamicin to determine survival **(B)**. For persistence, the bacteria were incubated for 23 h in gentamicin **(C)**. Cells were then lysed, and the extracts plated onto LB. *E. coli* HB101 was used as a negative control. The values shown are means plus standard deviations. Significant differences are indicated by asterisks (^***^P < 0.0001). At 4 h post-infection, the association of bacteria with macrophages was visualized by Giemsa staining **(D)**. Arrows indicate bacteria associated with the macrophages. Magnification is 1000×.

We next determined the impact of *fnr* mutation on the survival and persistence of bacteria in cultured macrophages using the J774 cell line (Figures 3A–D). Cells were infected with the WT, the *fnr* mutant, the complemented (pGEN-FNR) and *fnr* mutant containing the empty vector (pGEN-MCS) strains. Results showed deletion of *fnr* significantly decreased APEC O1's survival and persistence in macrophages (*P* < 0.01; Figures 3B,C) at even higher fold changes than seen with epithelial cells. Whereas, the WT strain displayed a survival of 1.05 bacteria per J774 cell, the *fnr* mutant displayed a survival rate of 0.009 bacteria per cell after 4 h of infection, a 116-fold reduction as compared to the WT. Similarly, the WT persisted at a rate of 0.14 bacteria per cell, while the *fnr* mutant persisted at a rate of 0.0001 bacteria per cell. Thus, the mutant strain showed a 1,400-fold reduction in persistence in J774 cells as compared to the WT. Also, reintroduction of *fnr* back into the mutant restored WT levels of survival and persistence in macrophage cells (Figures 3C,D). These results suggest that the *fnr* mutation impairs APEC O1's survival and persistence in macrophages.

### *In silico* prediction of FNR binding sites

Using an *in silico* analysis we found. Five hundred and sixteen FNR binding sites were predicted on the APEC O1 chromosome, and 19 binding sites were predicted on APEC O1's virulence plasmid, pAPEC O1-ColBM. Predicted binding sites were found upstream of most known virulence genes and operons, including plasmid genes such as those encoding *AatA*, a putative macrolide efflux pump, resistance to oxidative stress and *OmpT*, and encoded in the chromosome Type I Fimbriae, *ChuA* (heme receptor) and T6SS (Table 1). These seven genes chosen for study based on demonstration of gain in virulence in APEC or other pathogenic *E. coli* (Schlosser-Silverman et al., 2000; Johnson et al., 2006; Sabri et al., 2006; Johnson and Nolan, 2009; Li et al., 2009; de Pace et al., 2010; Gao et al., 2012; Thomassin et al., 2012) and the effects of FNR on their expression could be measured *in vitro*.

### Deletion of *fnr* reduced the expression of type 1 fimbriae in APEC

**Figure 4:**
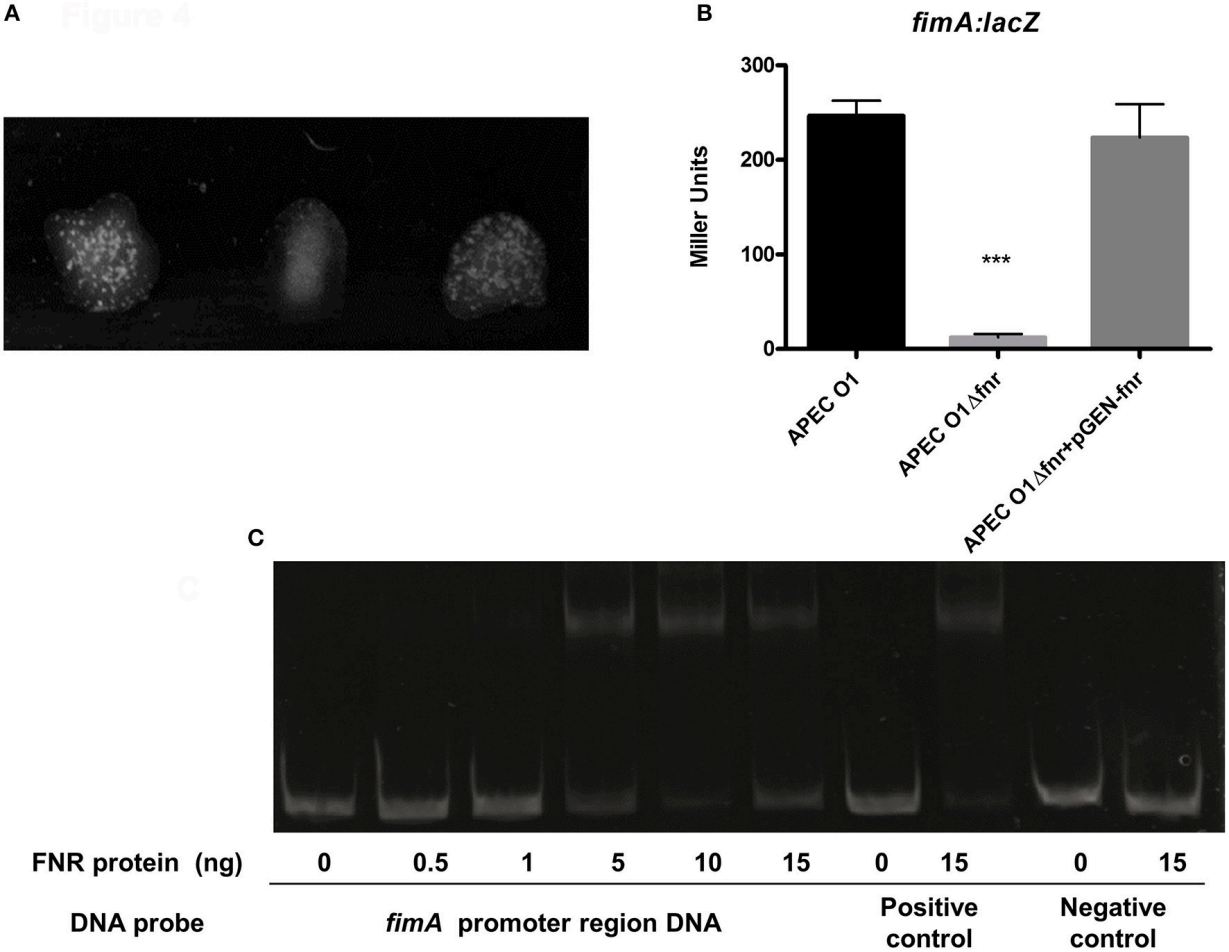
APEC type I fimbriae regulation. **(A)** Yeast agglutination assay with dilutions of WT 1:8; Δ*fnr* 1:1 and Δ*fnr* + pGEN-*fnr* 1:8. Bacteria were grown statically for 48 h, agglutination was read after 10 min at room temperature, and the strength of the agglutination was determined by titering of serial 2-fold dilutions of the bacterial suspension. Experiments were performed four times in quadruplicates. **(B)** β-galactosidase activity assay for expression of *fimA*. *fimA*-lacZ transcriptional fusion strains were grown in LB medium statically for 48 h at 37°C. β-galactosidase activity was measured, and the values shown as means plus standard deviations of triplicate samples from three independent experiments. Significant differences are indicated by asterisks (^***^P < 0.0001 compared to the WT and mutant). **(C)** Non-radioactive EMSA of binding of (fnrD154A)_2_—His6 to the promoter regions. PCR product of *fimA* promoter region was used as probes at 300 ng per each reaction. Purified (fnrD154A)_2_—His6 fusion protein was added at different concentrations in each reaction mixture as indicated; ydfZ promoter region DNA probes with and without FNR protein were used as positive controls; and *fimA* coding region DNA probes with and without FNR protein were used as negative controls. DNA fragments were stained with SYBR green.

The expression of type 1 fimbriae can be tested using a mannose-sensitive yeast agglutination assay, which assesses the ability of bacteria to bind to mannose receptors on the yeast cell surface. Using a serial dilution assay, we observed that wild-type APEC O1 agglutinated yeast cells at an 8-fold greater dilution than the APEC O1 *fnr* mutant. However, complementation of the mutant with pGEM—FNR restored wild-type levels of agglutination (Figure 4A). These results indicate that the *fnr* mutation caused a substantial decrease in the expression of type 1 fimbriae on the cell surface of APEC.

To provide further confirmation that this phenotypic change was due to regulation of the type 1 fimbriae genes, we evaluated expression of *fimA*, which encodes the major subunit of type 1 fimbriae, using β-galactosidase assays. Here, *fim-lacZ* constructs were created in the APEC O1 chromosome. A significant down-regulation of *fimA* was observed in the *fnr*-deletion mutant as compared with the wild type (Figure 4B), suggesting that FNR enhances type 1 fimbriation at the transcriptional level, a finding consistent with our agglutination data.

To determine whether FNR directly regulates *fimA* expression, an electrophoretic mobility shift assay (EMSA) was performed. DNA fragments containing the potential binding site were then amplified by PCR for use as probes. As shown in Figure 4C, the FNR fusion protein was able to shift the promoter fragment of *fim*. These results demonstrate that FNR directly binds to the promoter of the *fim* operon.

### Deletion of *fnr* reduced expression of AatA in APEC O1

**Figure 5:**
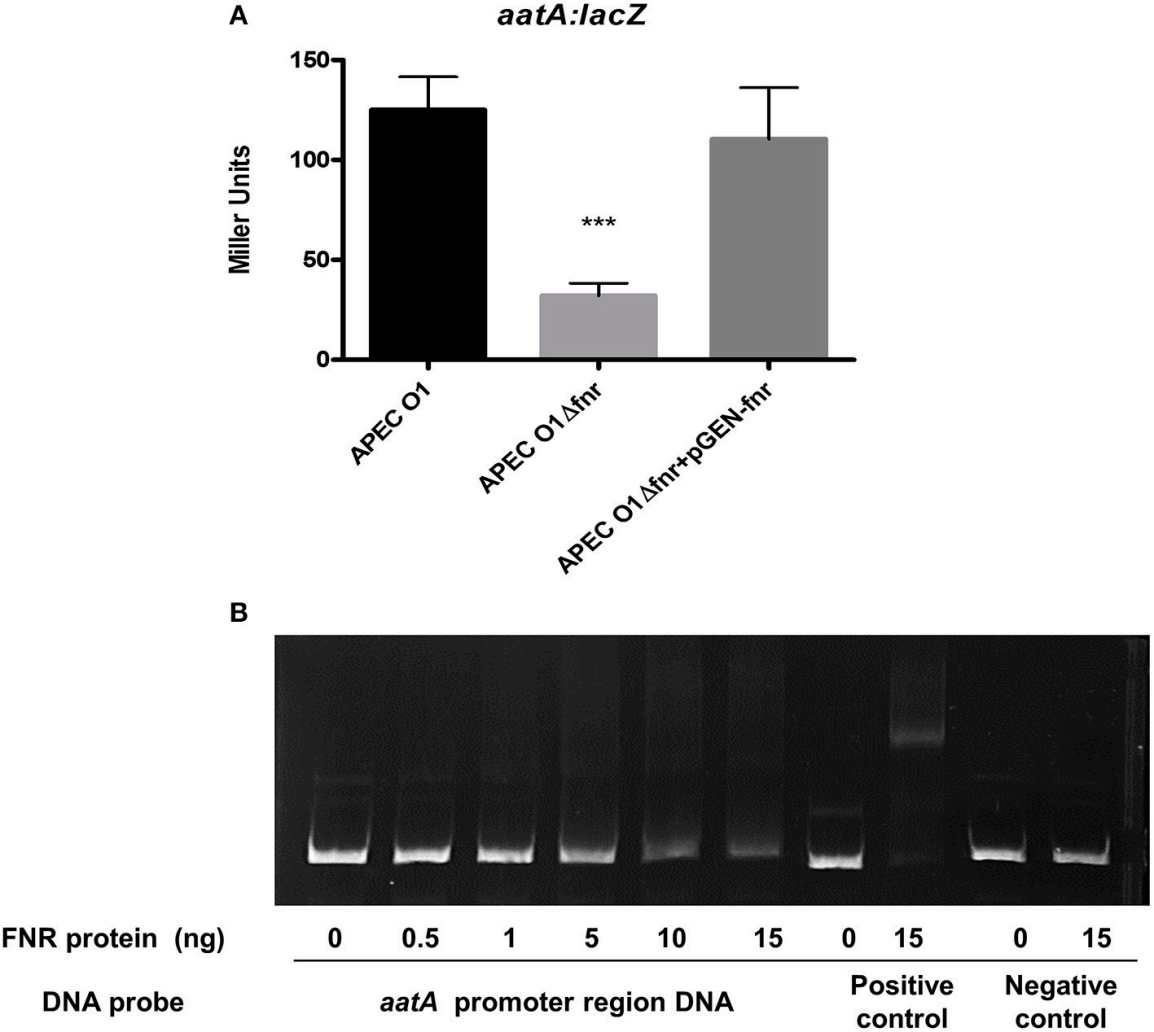
Regulation of AatA by FNR. **(A)** aatA-lacZ fusion strains were grown in LB until OD 0.5 at 37°C. β-galactosidase activity was measured, and the values shown are means plus standard deviations. Significant differences are indicated by asterisks (^***^P < 0.0001 compared to the WT and mutant). **(B)** Non-radioactive EMSA of binding of (fnrD154A)_2_—His6 to the promoter regions. PCR product of *aatA* promoter region was used as probes at 300 ng per each reaction. Purified (fnrD154A)_2_—His6 fusion protein was added at different concentrations in each reaction mixture as indicated; ydfZ promoter region DNA probes with and without FNR protein were used as positive controls; and *aatA* coding region DNA probes with and without FNR protein were used as negative controls. DNA fragments were stained with SYBR green.

Our group recently described an AatA autotransporter system in APEC O1 (APECO1_O1CoBM96) that contributes to APEC virulence *in vivo* (Li et al., 2009). A potential binding site of FNR in the promoter region of the *aatA* gene was identified by bioinformatic analysis. To determine if deletion of *fnr* affects *aatA* expression, we tested the gene expression by β-galactosidase assays. We found that *aatA* expression was significantly reduced (*P* < 0.001) in the *fnr* mutant as compared to the wild-type strain, and its expression was restored in the complemented strain to the wild-type level (Figure 5A).

To determine whether FNR directly regulates *aatA* expression, an EMSA was performed. DNA fragments containing the potential binding site were then amplified by PCR for use as probes. As shown in Figure 5B, the FNR fusion protein was able to shift the promoter fragment of *aatA* demonstrating that FNR directly binds to the promoter of *aatA*.

### FNR-deletion mutants show reduced expression of *ChuA*

**Figure 6:**
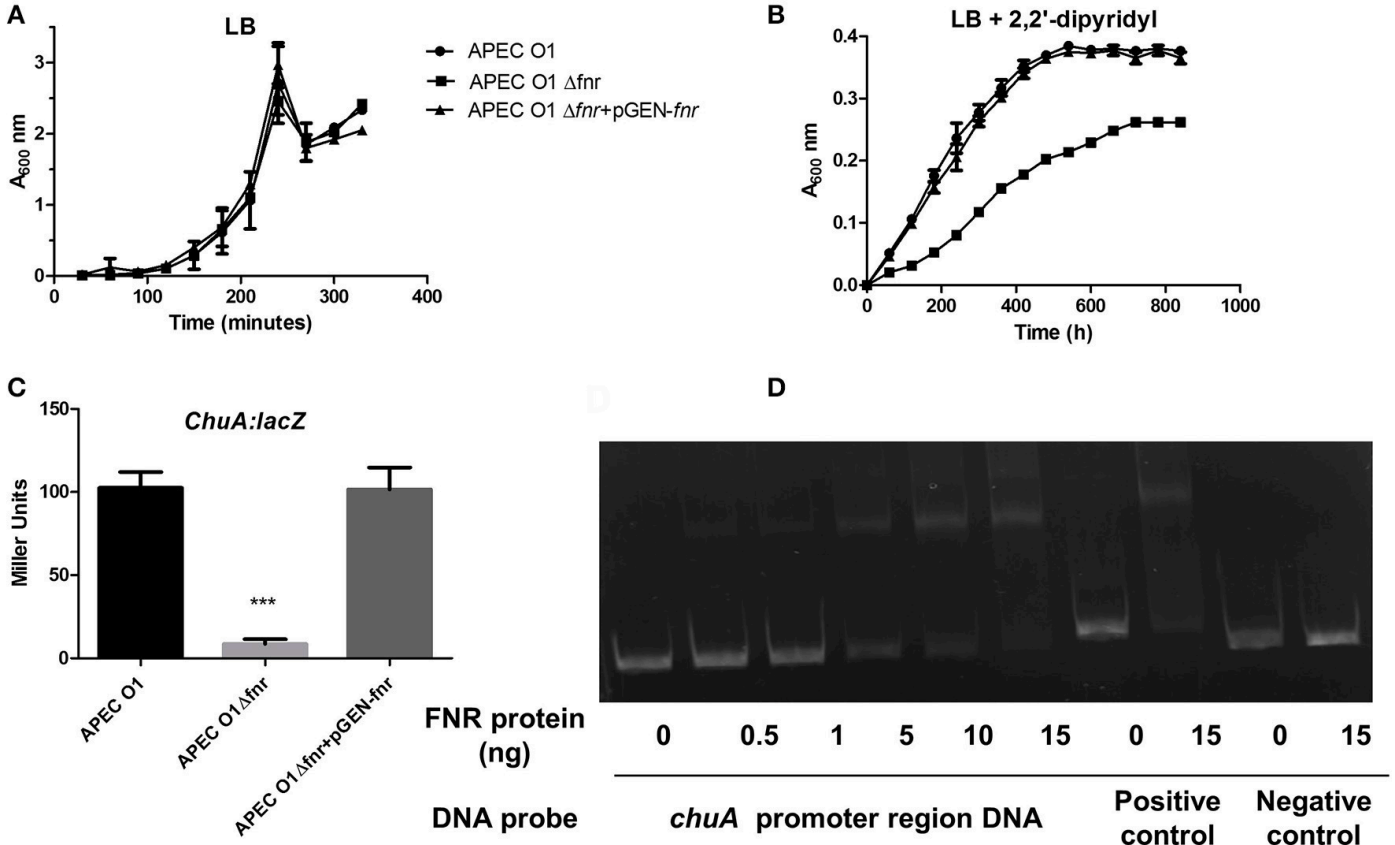
Regulation of ChuA by FNR. The optical density of the WT and mutants during growth in LB medium **(A)** or LB media with 12.5 μM 2.2 Dipyridyl **(B)**. Growth curves represent the average measurement at each time point in duplicate from three independent experiments. **(C)** *chuA*-*lacZ* fusion strains were grown in LB until OD 0.5 at 37°C. β-galactosidase activity was measured, and the values shown are means plus standard deviations. Significant differences are indicated by asterisks (^***^P < 0.0001 compared to the WT and mutant). **(D)** Non-radioactive EMSA of binding of (fnrD154A)_2_—His_6_ to the promoter regions of *chuA* was performed as described for Figure 4. The PCR product of *chuA* promoter region was used as probe.

For investigation of *chuA* expression, we grew the FNR mutant and the wild-type (APEC O1) strains under iron-deficient conditions using media containing an iron chelator (Figures 6A,B). We compared the WT, ΔFNR mutant, and the pGEN-FNR mutant complemented for ability to grow under iron-deficient conditions. The FNR mutants showed reduced capability to grow in the iron-deficient medium. Such results suggest that expression of *chuA* is important for iron acquisition in *E. coli* and that *chuA* is an important APEC O1 virulence factor (Payne, 1993).

To determine if deletion of FNR affects *chuA* expression, β-galactosidase assays were performed as shown in Figure 6C as described previously (Barbieri et al., 2014). These results demonstrate that expression of *chuA* was significantly reduced in the FNR mutant as compared to the wild type (Figure 6C). EMSAs confirmed that direct regulation of *chuA* by FNR occurred (Figure 6D).

### FNR-deletion mutants show a reduction in of T6SS *vgrG*

**Figure 7:**
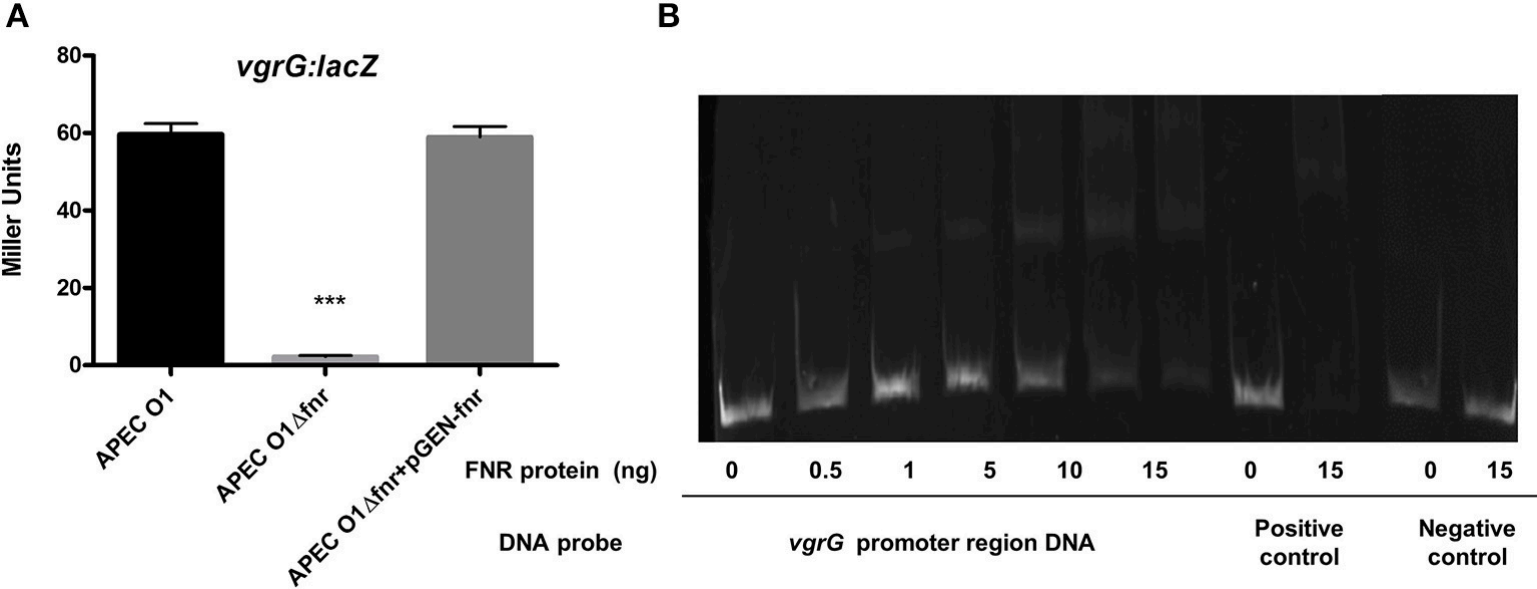
Regulation of T6SS by FNR. **(A)** *vgrG*-*lacZ* fusion strains were grown in LB overnight at 37°C. β-galactosidase activity was measured, and the values shown are means plus standard deviations. Significant differences are indicated by asterisks (^***^P < 0.0001 compared to the WT and mutant). **(B)** Non-radioactive EMSA of binding of (fnrD154A)_2_—His_6_ to the promoter regions of *vgrG* was performed as described for Figure 4. The PCR product of *vgrG* promoter region was used as probe.

The amoeba *Dictyostelium* was used to demonstrate the phenotype of the type 6 secretion system. We observed that more amoeba cells were required to kill WT APEC O1 when compared to FNR mutants (Table 2). Further, the expression of *vgrG* was significantly reduced in the *fnr* mutants as compared to the wild type (Figure 7A). EMSA results confirmed that direct regulation of *vgrG* by FNR occurred (Figure 7B).

**Table 2:** FNR contributes to *E. coli* survival in Dictyostelium assay.

**Strain**	**Dictyostelium concentration (cells/spot)**
APEC O1	5 × 10^3^
APEC O1 Δ*fnr*	5 × 10^1^
APEC O1 Δ*fnr* +pGEN-*fnr*	5 × 10^3^

### FNR-deletion mutants show reduced action in putative macrolide efflux by *ets*

**Figure 8:**
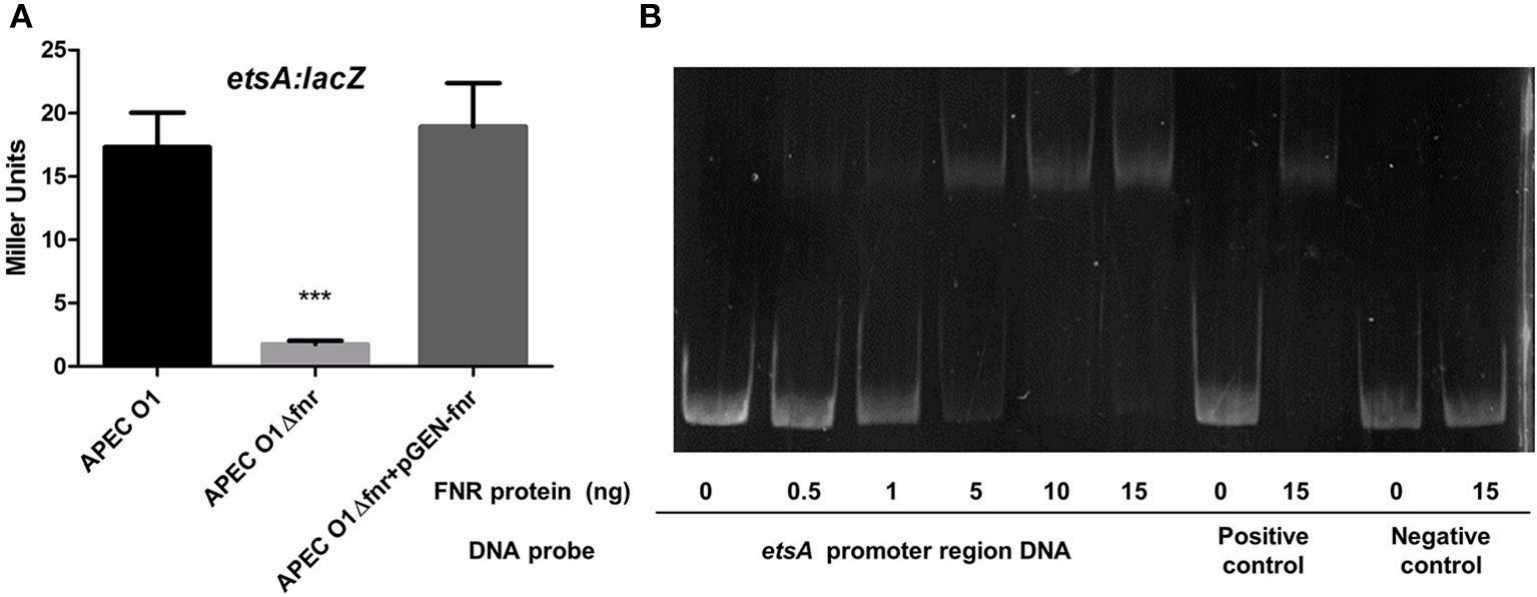
Regulation of etsA by FNR. **(A)** *etsA*-*lacZ* fusion strains were grown in LB overnight at 37°C. β-galactosidase activity was measured, and the values shown are means plus standard deviations. Significant differences are indicated by asterisks (^***^P < 0.0001compared to the WT and mutant). **(B)** Non-radioactive EMSA of binding of (fnrD154A)_2_—His_6_ to the promoter regions of *etsA* was performed as described for Figure 4. The PCR product of *etsA* promoter region was used as probe.

To determine if FNR deletion affects expression of the putative EtsA macrolide efflux pump, we used β-galactosidase assays. We found that the FNR mutant was 4-fold more susceptible to erythromycin than the wild-type strain (Table 3). Here, we found that expression of the EtsA system was significantly reduced in the FNR mutant as compared to the wild type (Figure 8A). EMSAs confirmed that FNR directly regulates *etsA* expression (Figure 8B).

**Table 3:** FNR contributes to Erythromycin resistance.

**Strain**	**MIC μg/ml**
	**Erythromycin**
APEC O1	128
APEC O1 Δ*fnr*	32
APEC O1 Δ*fnr* +pGEN-*fnr*	128
MG1655	32

### FNR-deletion mutants showed reduced expression of *mig-14*

**Figure 9:**
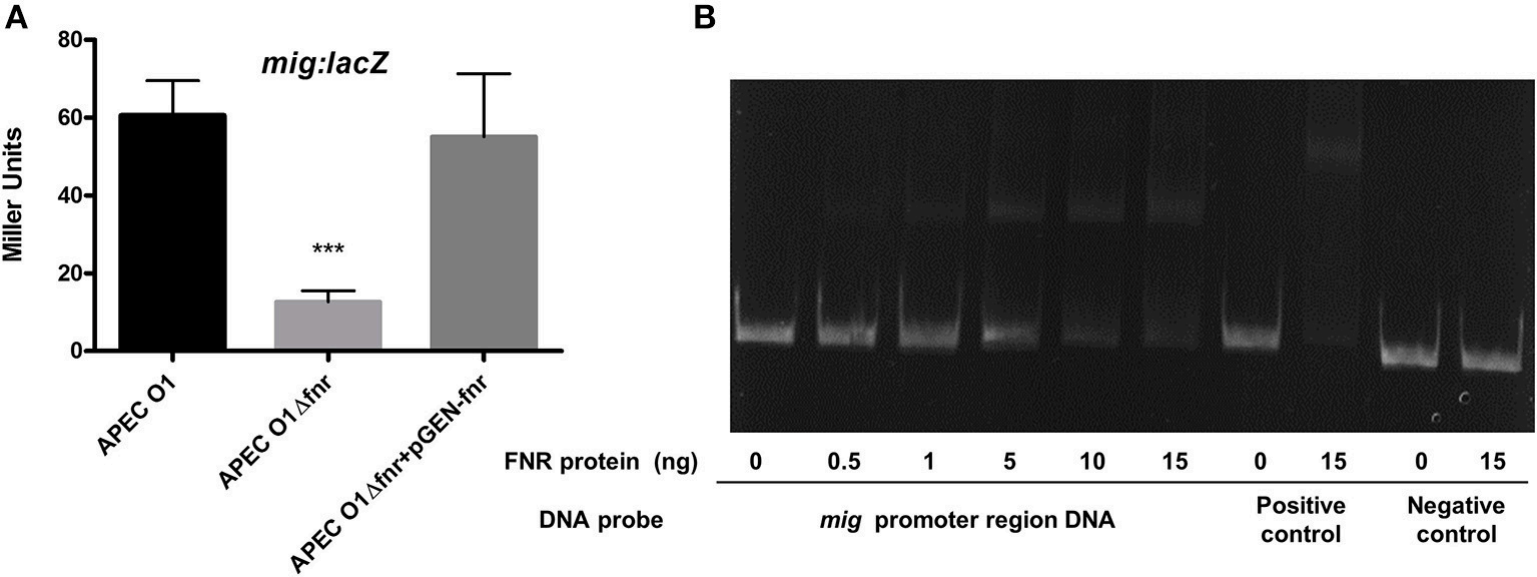
Regulation of mig-14 by FNR. **(A)** *mig*-*lacZ* fusion strains were grown in LB overnight at 37°C. β-galactosidase activity was measured, and the values shown are means plus standard deviations. Significant differences are indicated by asterisks (^***^P < 0.0001 compared to the WT and mutant). **(B)** Non-radioactive EMSA of binding of (fnrD154A)_2_—His_6_ to the promoter regions of *mig* was performed as described in Figure 4. The PCR product of *mig* promoter region was used as probe.

FNR mutants were shown to have reduced ability to overcome oxidative stress (Table 4). This change in phenotype has real world significance, as resistance to oxidative stress is critical to bacterial survival when they are exposed to the respiratory burst, following phagocytosis, which may result in bacterial cell death. Our results using β-galactosidase assays show that expression of *mig*-*14* was reduced by FNR mutation (Figure 9A). Further, EMSAs confirmed that FNR directly regulates expression of *mig-14* (Figure 9B).

**Table 4:** FNR contributes to oxidative stress.

**Strain**	**MIC**
	**H_2_O_2_ (%)**
APEC O1	1.87
APEC O1 Δ*fnr*	0.46
APEC O1 Δ*fnr* +pGEN-*fnr*	1.87

### FNR contributes to APEC's antimicrobial peptide resistance

It was previously reported that pathogenic *E. coli* strains are resistant to antimicrobial peptides such as LL-37, lysozyme, and polymyxin B (Thomassin et al., 2012). Here, we found that APEC O1 was resistant to the antimicrobial peptide LL-37, having a MIC of 128 μg/ml, as compared to the FNR mutant's MIC of 32 μg/ml, i.e., the mutant was 4-fold more susceptible to LL-37 peptide than the wild type (Table 5). Moreover, the wild-type level of resistance was restored when APEC O1 Δ*fnr* was complemented with pGEN-FNR, having an MIC of 128 μg/ml.

**Table 5:** FNR contributes to antimicrobial peptide resistance.

**Strain**	**MIC μg/ml**		
	**LL-37**	**Lysozyme**	**Polymyxin B**
APEC O1	128	2,500	4
APEC O1 Δ*fnr*	32	312	4
APEC O1 Δ*fnr* +pGEN-*fnr*	128	2,500	4
MG1655	32	312	4

We also tested the MIC in relation to lysozyme. Our results showed that APEC O1 had an MIC of 2,500 μg/ml and APEC O1 Δ*fnr* had an MIC of 312 μg/ml with wild type levels of resistance to lysozyme seen in the complemented mutant (APEC O1 Δ*fnr* + pGEN-FNR; Table 5). APEC O1 Δ*fnr* was 8-fold more susceptible to lysozyme, indicating that FNR is also important for resistance to lysozymal action.

However, no differences were seen between the wild type and its FNR mutant in resistance to the antimicrobial action of polymyxin B (Table 5). All strains showed a MIC of 4 μg/ml.

### Deletion of *fnr* reduced expression of the plasmid-linked *ompT* gene

**Figure 10:**
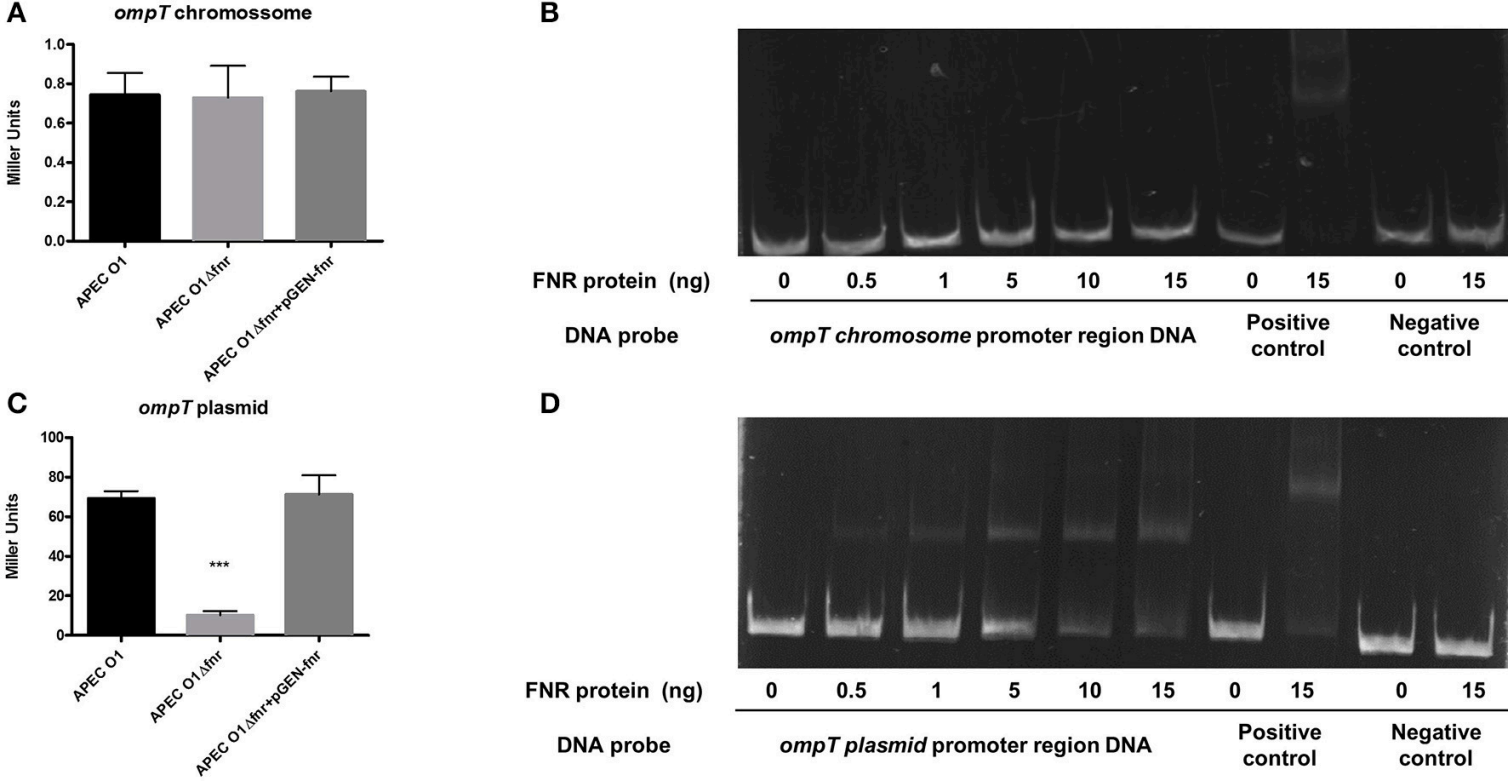
Regulation of OmpT by FNR. **(A)** *ompTchr*-*lacZ* and **(C)** *ompTplas*-*lacZ* transcriptional fusion strains were grown in LB until OD 0.5 at 37°C. β-galactosidase activity was measured, and the values shown are means plus standard deviations. Significant differences are indicated by asterisks (^***^P < 0.0001 compared to the WT and mutant). Non-radioactive EMSA of binding of (fnrD154A)_2_—His_6_ to the promoter regions. PCR product of *ompT chr* **(B)** *and ompT plas* **(D)** Non-radioactive EMSA of binding of (fnrD154A)_2_—His_6_ to the promoter regions of *ompT* was performed as legend of Figure 4. PCR product of *ompT* promoter region was used as probe.

*ompT* is an important gene for resistance to antimicrobial peptides (Thomassin et al., 2012). In the APEC O1 genome, two copies of the *ompT* gene exist. One copy is in the chromosome (APECO1_1482), and the second is located on the pAPEC O1-ColBM virulence plasmid (APECO1_O1CoBM192). Through *in silico* prediction, we observed an FNR binding motif in the promoter region of the plasmid-located *ompT* but not in the chromosomal *ompT*. We constructed *ompT-lacZ* fusions to test expression of the chromosome and plasmid *ompT* genes independently. Expression of the chromosomal *ompT* gene was not altered with *fnr* mutation (Figure 10A). However, expression of the plasmid-encoded *ompT* gene was significantly reduced in the *fnr* mutant (Figure 10C), a reduction that was restored to wild-type levels in the *fnr* complemented strain.

To determine whether FNR directly regulates *ompT* expression, an EMSA was performed. A potential binding site of FNR was identified by bioinformatic analysis. DNA fragments containing the potential binding site were then amplified by PCR for use as probes. As shown in Figure 10B, FNR did not shift the promoter fragment of the chromosomal *ompT*, but it did directly bind to the plasmid-located *ompT* regulatory region (Figure 10D).

## Discussion

We have linked FNR to the regulation of APEC O1 virulence genes by the binding of the FNR protein to chromosomal and plasmid DNA under aerobic conditions. This finding is important because FNR is recognized as a transcription factor that is a global regulator of pathways meant to facilitate gene expression (Kiley and Beinert, 1999; Barbieri et al., 2014). Although FNR's role in regulation of *E. coli*'s gene expression has been documented (Guest et al., 1996; Shan et al., 2012; Barbieri et al., 2014), this research is the first to show the global impact of FNR regulation on the virulence of an APEC strain.

FNR is related to transcriptional activators that control the expression of networks of *E. coli* genes in response to oxygen starvation, as observed in non-pathogenic *E. coli* strains such as *E. coli* K-12 (MG 1655; Guest et al., 1996). Previous studies, under anaerobic conditions, FNR was able to bind to specific DNA targets at promoter sites and modulate transcription (Kiley and Beinert, 2003; Kang et al., 2005; Salmon et al., 2005; Anjum et al., 2016), however here we found that FNR were able to regulate APEC virulence genes under aerobic conditions.

By bioinformatic analysis, we identified 516 FNR binding sites in the APEC O1 chromosome and 19 binding sites in the plasmid pAPEC O1-ColBM. Among these were genes related to metabolic functions, transport of small molecules, iron metabolism, regulatory genes and genes related to outer membrane protein production. It is interesting that some of the genes targeted for regulation have no apparent connection to bacterial survival during the transition from aerobic to anaerobic environments, leading us to believe that *fnr* is more than what it is commonly thought to be, just an oxygen sensor (Shan et al., 2012), but also an important player in APEC virulence.

OmpT is a protease (Rodriguez-Siek et al., 2005) that is associated with the degradation of α-helical antimicrobial peptides. Such peptides are usually small cations that are secreted into the extracellular environment by epithelial cells and have both bactericidal and immunomodulatory properties. These peptides are key players in the innate immune response to infection. They bind to the anionic cell membrane of bacterial cells and lyse them through pore-forming action (Thomassin et al., 2012). They also act as a bridge between innate and adaptive immunity by recruiting immune cells to the site of infection (Thomassin et al., 2012). LL-37 is one example of an antimicrobial peptide; it is expressed by different cell types, including neutrophils, bone marrow cells and epithelial cells of the lung and intestine (Nijnik and Hancock, 2009). LL-37's biological precursor is human cationic antimicrobial protein 18 (hCAP18), which is processed into the biologically active peptide by serine protease proteinase 3 (Nijnik and Hancock, 2009; Thomassin et al., 2012). Previous reports showed that OmpT of Enterohemorrhagic *E. coli* (EHEC) readily degraded and inactivated antimicrobial peptides to promote bacterial survival; whereas, OmpT of Enteropathogenic *E. coli* (EPEC) plays a marginal role in antimicrobial peptide degradation (Thomassin et al., 2012). Proteolytic degradation of α-helical antimicrobial peptides, such as LL-37, may be accomplished by outer membrane proteases, which has shown to be the case in Gram-negative pathogens (Thomassin et al., 2012). As we observed by bioinformatic analysis, the *ompT* present in the pAPEC O1-ColBM plasmid has an FNR binding motif in its promoter region. We observed that plasmid located *ompT* was down-regulated in the *fnr* mutant strains (Figure 10C) compared to the wild type. In contrast, chromosomal *ompT* expression was not altered in the mutant strain (Figure 10A). These data suggest that the observed resistance to antimicrobial peptides in APEC O1 is, indeed, due to FNR gene regulation of the plasmid-located *ompT* gene. This finding is novel, as *ompT*'s role in the pathogenesis of avian colibacillosis has been unclear (Rodriguez-Siek et al., 2005).

Also, using an EMSA assay, we confirmed that only the plasmid-located copy of *ompT* bound the FNR protein. These data clearly indicate that FNR only regulates one of two copies of the same gene. We also found that the plasmid and chromosomal versions of *ompT* employ two different promoter regions. Based on these observations, we hypothesize that different environmental cues stimulate chromosomal and plasmid *ompT* transcription and that the two OmpTs may have different biological functions as well.

Antimicrobial peptides are an important new family of molecules developed for treatment of infection by multi-resistant bacteria. Here, we tested APEC O1's susceptibility to LL-37, lysozyme, and polymyxin B (Table 5). We found that wild-type APEC O1 was more resistant to LL-37 than its FNR mutant. The enhanced susceptibility was owed to a gene regulated by FNR unrelated to its oxygen sensor activity but related to APEC O1's metabolic abilities, as previously described (Kiley and Beinert, 2003).

AatA is an autotransporter encoded by the APEC gene *aatA*, which has been localized to the PAI found in the virulence plasmid pAPEC-O1-ColBM of APEC O1 (Li et al., 2009). *aatA* is present in 32–40% of APEC isolates (Li et al., 2009; Dai et al., 2010), and the protein it encodes, AatA, contributes to adherence (Dai et al., 2010) and virulence of APEC O1 *in vivo* (Li et al., 2009). Here, we showed that *aatA* was upregulated by FNR. Further, we were able to demonstrate that FNR binds to the promoter region of the *aatA* gene (Figure 5), enabling direct regulation by FNR.

Efflux pumps are encoded by specific genes in *E. coli* and could be under the control of a transcription factor such as FNR (Rosenberg et al., 2003). Further, investigation of erythromycin susceptibility was accomplished by MIC assays using erythromycin, a macrolide commonly used in poultry production for treatment of colibacillosis. Efflux pumps aid *E. coli* in resisting the negative effects of antimicrobial agents by pumping the noxious substance out of the bacterial cell. It is thought that *etsA* could be an important aid for efflux action to rid the bacteria of macrolides such as erythromycin, clarithromycin, and azithromycin (Rosenberg et al., 2003; Johnson et al., 2006). Based on our results, we conclude that the EtsA putative efflux pump is under control by FNR.

The presence of large virulence plasmids typify APEC strains (Johnson et al., 2010, 2012) and contribute in multiple ways to their ability to cause disease (Johnson et al., 2006; Johnson and Nolan, 2009). Such plasmids are often transferable to other bacterial strains via conjugation (Wooley et al., 1992), where they enhance a recipient's ability to exploit host environments and cause disease (Mellata et al., 2010). This report is the first time that FNR, a protein encoded by the chromosome, is reported to regulate genes present on plasmids, such as the plasmid-located *ompT, aatA, etsA*, and *mig-14* genes.

Type 1 fimbriae are important adhesins of APEC strains and are important to the pathogenesis of avian colibacillosis (La Ragione et al., 2000; de Pace et al., 2010; Chanteloup et al., 2011). Type I fimbriae, which are present in almost all APEC isolates (Ewers et al., 2007; Barbieri et al., 2013), are one of the most common virulence-associated factors in these pathogens. Here, we observed that the *fnr* mutant was unable to agglutinate yeast cell extracts at a high concentration. Further, we observed that *fimA* expression was reduced in the mutant strain (Figure 4). These findings agree with our previous work with Uropathogenic *E. coli* (Barbieri et al., 2014) and further confirms that *fnr* mutants of APEC O1 are less able to express type I fimbriae than their wild-type counterpart. Previous work has shown that the loss of type I fimbriae causes a reduction of adhesion to human pneumocytes (A549) and hepatocytes (LM; Chanteloup et al., 2011), reduction of biofilm formation (Anderson et al., 2003), reduction in bacterial adhesion to eukaryotic cells (La Ragione et al., 2000), and reduction in pathogenicity *in vivo* (Marc et al., 1998). The FNR regulation of type I fimbriae expression underscores that type I fimbriae is an important virulence-associated gene in APEC.

Most iron in the vertebrate host is bound in complexes, such as the heme complex, making it unavailable to bacterial pathogens. Thus, the ability to acquire iron in the host environment during infection is critical to the virulence of *E. coli* (Barbieri et al., 2014). Reflecting the importance of iron during infection, APEC encodes many mechanisms to manage its need for iron (Sabri et al., 2006). ChuA is an outer membrane protein responsible for heme uptake in *E. coli* (Nagy et al., 2001). Thus, regulation of ChuA's activity would seem to be important in *E. coli*'s pathogenesis.

Another candidate target of FNR regulation is T6SS, a recently discovered component of Gram-negative bacteria that interacts with both prokaryotic and eukaryotic cells (Silverman et al., 2012). The T6SS contributes to several different functions including eukaryotic cell targeting, inter-bacterial cell targeting, and cellular adhesion (Zheng et al., 2011; Silverman et al., 2012). Functionally, the T6SS mimics T4 bacteriophages by forming needle-like structures that pierce other cells and form channels between bacteria and target cells allowing for secretion of proteins, attachment, and other functions (Shrivastava and Mande, 2008). The amoeba *Dictyostelium* spp. can be used to demonstrate expression of the T6SS (Zheng et al., 2011). Pukatzki *et al*. have shown that *Vibrio cholerae* strain V52 was able to kill the amoebae due to a gene cluster that encodes a distinct secretion system, which they designated T6SS (Pukatzki et al., 2006). The VgrG subunit contributes to the formation of the needle and is likely released from the system after the target cell is punctured (Silverman et al., 2012). The T6SS has been associated with pathogenesis of diverse bacterial species, including the pathogenicity of APEC strains (de Pace et al., 2010). Here, our findings reinforce the importance of T6SS in APEC virulence.

Here, we describe FNR's regulation of APEC virulence-associated genes, *ompT, etsA*, mig-14, and *aatA* under aerobic conditions. Indeed, all of these plasmid-linked virulence-associated genes are under FNR control for expression with reduced expression found in FNR mutants using β-galactosidase assays.

In summary, our results show that FNR in APEC O1 is not just an oxygen sensor, but also an important regulator of virulence-associated genes in APEC under aerobic conditions.

## Author contributions

Conceived and designed the experiments: NB, GL, and LN. Performed the experiments: NB, JV, and AB. Analyzed the data: NB, GL, CL, and LN. Contributed reagents/materials/analysis tools: FH, CL, and LN. Wrote the paper: NB, GL, CL, and LN.

### Conflict of interest statement

The authors declare that the research was conducted in the absence of any commercial or financial relationships that could be construed as a potential conflict of interest.
