# Supplementary material for: FNR Regulates the Expression of Important Virulence Factors Contributing to the Pathogenicity of Avian Pathogenic Escherichia coli
Source: Front Cell Infect Microbiol. 2017 Jun 23;7:265. doi: 10.3389/fcimb.2017.00265 (PMC5481319; doi:10.3389/fcimb.2017.00265)
Supplement: Supplementary file 1 [file Table1.DOCX]

**Table S1. Strains and plasmids.** The genotypes of all strains of *E. coli* used or constructed in this study and information about the plasmids used in this study.

| **Bacterial strains and plasmids** | **Genotype or relevant characteristics** | **Source or Reference** |
| --- | --- | --- |
| *E. coli* MG1655 | K-12 strain | Invitrogen |
| *E. coli* HB101 | K-12 strain | (9) |
| *E. coli 23558* | Colicin V positive control | (38) |
| *E. coli 23559* | Colicin V negative control | (38) |
| *E. coli* S17-λpir | RK2 tra regulon, pir, host for pir-dependent plasmids | (60) |
| *E. coli*  fnr-771(del)::kan | F-, Δ(araD-araB)567, ΔlacZ4787(::rrnB-3), λ-, Δfnr-771::kan,rph-1, Δ(rhaD-rhaB)568, hsdR514 | (61) |
| APEC O1 | isolate from lesions of chickens clinically diagnosed with colibacillosis | (14) |
|  | APECO1 ∆*lacZYA* | This study |
|  | APECO1 ∆l*acZYA*::Chlr | This study |
|  | APECO1 ∆*fnr* | This study |
|  | APECO1 ∆*fnr*::kan | This study |
|  | APECO1 ∆l*acZYA* ∆*fnr* | This study |
|  | APECO1 ∆lacZYA fimA-lacZ | This study |
|  | APECO1 ∆lacZYA ∆fnr fimA-lacZ | This study |
|  | APECO1 ∆lacZYA aatA-lacZ | This study |
|  | APECO1 ∆lacZYA ∆fnr aatA-lacZ | This study |
|  | APECO1 ∆lacZYA ompTchr-lacZ | This study |
|  | APECO1 ∆lacZYA ∆fnr ompTchr-lacZ | This study |
|  | APECO1 ∆lacZYA ompTplas-lacZ | This study |
|  | APECO1∆lacZYA ∆fnr ompTplas-lacZ | This study |
|  | APECO1 ∆*lacZYA chuA::lacZ* | This study |
|  | APECO1 ∆*lacZYA ∆fnr chuA::lacZ* | This study |
|  | APECO1 ∆*lacZYA etsA::lacZ* | This study |
|  | APECO1 ∆*lacZYA ∆fnr etsA::lacZ* | This study |
|  | APECO1 ∆*lacZYA mig::lacZ* | This study |
|  | APECO1∆*lacZYA ∆fnr mig::lacZ* | This study |
| **Plasmids** |  |  |
| pET28a-(fnrD154A)2 | expression plasmid of FNR protein | (30) |
| pVIK112 | suicide plasmid for chromosomal lacZ transcriptional fusion | (24) |
| pGEN-MCS | low copy plasmid for complementation | (25) |
| pGEM-FNR | pGEN-MCS carrying fnr coding region and 500bp upstream promoter region | This study |
| pKD3 | template for λ-Red Chl^r^ cassette | (62) |
| pKD4 | template for λ-Red Kan^r^ cassette | (62) |
| pCP20 | encodes FLP recombinase for removal of resistance cassette | (62) |
| pKD46 | λ-Red recombinase expression | (62) |
