# Supplementary material for: FNR Regulates the Expression of Important Virulence Factors Contributing to the Pathogenicity of Avian Pathogenic Escherichia coli
Source: Front Cell Infect Microbiol. 2017 Jun 23;7:265. doi: 10.3389/fcimb.2017.00265 (PMC5481319; doi:10.3389/fcimb.2017.00265)
Supplement: Supplementary file 2 [file Table2.DOCX]

**Table S2. Oligonucleotides.** Oligonucleotide sequences used as PCR primers.

| **Primers** | **Sequence (5'-3')** |
| --- | --- |
| ***General PCR for cloning*** |  |
| pGEN-FNR-F | GTACCATATGGATCGAATCCCATCAGCATC |
| pGEN-FNR-R | CCGAGTCGACAGGATCGATAACAACGAGCA |
| ***For lacZ fusion*** |  |
| fimA-lacZ-5'-F | CACGGAATTC GTTGATGCAGGCTCTGTTGA |
| fimA-lacZ-5'-R | GCAGTCTAGA TTATTGATACTGAACCTTGA |
| ompTgen-lacZ-5'-F | CACGGAATTCATTGCGAGGCCTTATGTGTC |
| ompTgen-lacZ-5'-R | GCAGTCTAGAGCCTCCTTCTTCGGCTAGAT |
| ompTplas-lacZ-5'-F | GACGGAATTCGGCCTTATTCGCAGTTACCA |
| ompTplas-lacZ-5'-R | GTCGTCTAGATCAGGCAGATAAACCCGTTC |
| aatA-lacZ-5'-F | CACGGAATTCGGGAATATCTACGGCAGCAA |
| aatA-lacZ-5'-R | GCAGTCTAGATGCACCACCGATTGATGTAT |
| chuA-lacZ-5'-F | cacgGAATTCtaagggaaaacgcacagcac |
| chuA-lacZ-5'-R | gcagTCTAGAaaagcaaacgttggcaaggt |
| vgrG-lacZ-5'-F | cacgGAATTC aaggttgagtgggagcacat |
| vgrG-lacZ-5'-R | gcagTCTAGA cgtcaacctccagcgtaaag |
| etsA-lacZ-5'-F | cacgGAATTCgagggggttagcccaattta |
| etsA-lacZ-5'-R | gcagTCTAGAttacccgttcaattgcatca |
| mig-lacZ-5'-F | cacgGAATTCtccctgtaacgaacgcctta |
| mig-lacZ-5'-R | gcagTCTAGAttccaggccgtaataatgcg |
| ***For Deletion^a^*** |  |
| Del-fnr-F | GACGGTTATGCCAGACCACT |
| Del-fnr-R | AAGCGACAAGCTTCGTGAAT |
| ***For EMSA*** |  |
| Inside negative control-For1 | ATCTGTGTGGTAAGAGAATC |
| Inside negative control-Rev1 | TGGTGCGCCATGGGATATTG |
| PromompTgen-For | TCAGGCAGATAAACCCGTTC |
| PromompTgen-Rev | AAACAAACGTTAAACAAAACAGCA |
| PromompTplas-For | AAACAAACGTTAAACAAAACAGCA |
| PromompTplas-Rev | TCAGGCAGATAAACCCGTTC |
| PromaatA-For | CCAGACGTCTCGTGATACTCC |
| PromaatA-Rev | CAGAGGCGTTCGAGCATTAT |
| Prom chu-For | atctccgggttctcagcttt |
| Prom chu-Rev | tggtgatgatccttggcata |
| Prom vgrG-For | gccggaacgattgtgaagta |
| Prom vgrG-Rev | tcaggaaccacacccttttc |
| Prom ets-For | GAGGGGGTTAGCCCAATTTA |
| Prom ets-Rev | TTCTGGCACATCAGAGCTTC |
| Prom mig-For | tgagtgtaagccctgactcaaa |
| Prom mig-Rev | agagcgtccagtctgcaaat |
| PromydfZ-For | GCGACTGGTTTAGCGAAGAG |
| PromydfZ-Rev | TGGTGATTGCGTTACGGTTA |

- Underlined are restriction cutting sites;
